# Single transcript level atlas of oxytocin and the oxytocin receptor in the mouse brain

**DOI:** 10.7554/eLife.95215

**Published:** 2026-02-23

**Authors:** Vitaly Ryu, Anisa Azatovna Gumerova, Georgii Pevnev, Funda Korkmaz, Hasni Kannangara, Liam Cullen, Farhath Sultana, Ronit Witztum, Steven Lee Sims, Tal Frolinger, Ofer Moldavski, Orly Barak, Emily Weiss, Jay J Cao, Daria Lizneva, Ki A Goosens, Tony Yuen, Mone Zaidi

**Affiliations:** 1 Institute for Translational Medicine and Pharmacology (ITMaP), Icahn School of Medicine at Mount Sinai New York United States; 2 Departments of Medicine and of Pharmacological Sciences, Icahn School of Medicine at Mount Sinai New York United States; 3 United States Department of Agriculture, Grand Forks Human Nutrition Research Center, Grand Forks, ND 58203 Grand Forks United States; Albert Einstein College of Medicine United States; State University of New York Upstate Medical University United States

**Keywords:** posterior pituitary hormones, spatial expression, in situ hybridization, oxytocin receptor, GPCR, Mouse

## Abstract

Oxytocin (OXT), a primitive nonapeptide known to regulate reproduction and social behaviors, is synthesized primarily in the hypothalamus and is secreted via the hypophyseal-portal system of the posterior pituitary gland. In line with the premise that pituitary hormones, traditionally thought of as regulators of single targets, display an array of central and peripheral actions, we found that OXT directly affects bone and body composition. The effect of OXT on bone remodeling is physiologically relevant, as elevated OXT levels during pregnancy and lactation cause calcium mobilization from the maternal skeleton for intergenerational calcium transfer towards fetal bone mineralization. There is an equally large body of evidence that has established the presence of OXT receptors (OXTRs) in the brain through which central functions, such as social bonding, and peripheral functions, such as the regulation of body composition, are exerted. To purposefully address effects of OXT on the brain, we used RNAscope to map OXT and OXTR expression, at the single transcript level, in the whole female and male mouse brains. Identification of brain nuclei with the highest OXT and OXTR transcript density sheds further light on functional OXT nodes that could be further interrogated experimentally to define new physiologic circuitry.

## Introduction

Oxytocin (OXT), a neuropeptide synthesized primarily by magnocellular neurons within the paraventricular (PVH) and supraoptic nuclei (SON) of the hypothalamus (Sofroniew, 1983; Swanson and Sawchenko, 1983; Landgraf and Neumann, 2004), has been broadly implicated in the control of parturition, lactation, appetite, emotions, stress responses, and social behavior. The distribution of OXT receptors (OXTRs) across the brain in different species provides a proxy for the distribution of OXT binding, thus providing evidence for OXT nodes in the brain of physiologic relevance. It has been reported that OXTRs are expressed in many brain sites, including the central nucleus of the amygdala and the ventromedial hypothalamic nucleus (VMH) (Bale and Dorsa, 1995b; Bale and Dorsa, 1995a). Furthermore, *Oxtr* mRNA has been detected in the hypothalamus, olfactory bulb, ventral pallidum, and the dorsal vagal nucleus (Yoshimura et al., 1993; Adan et al., 1995).

OXT mediates a variety of peripheral and central functions. While the peripheral actions comprise milk ejections, uterine contractions, and prolactin production, the central actions of OXT are mostly related to female reproduction, including sexual receptivity (Caldwell et al., 1986), pair bonding (Insel, 1992), and maternal behavior (Fahrbach et al., 1984; Pedersen et al., 1982; Insel, 1990). Central functions of OXT also include modulation of cardiac vagal input (Bohus et al., 1996), memory consolidation (Dyball and Paterson, 1983), and social/affiliative behavior (Insel, 1992; van Wimersma Greidanus and Maigret, 1996). Axons and dendrites of OXT neurons are localized in close proximity to the third ventricle and even in between tanycytes and ependymal cells facing the cerebrospinal fluid (Landgraf and Neumann, 2004). Notably, magnocellular OXT neurons send extended dendritic trees, forming the basis for the somato-dendritic release of OXT within the PVH and SON (Ludwig and Leng, 2006; Neumann et al., 1993; Neumann, 2007; Pow and Morris, 1989). Such release is likely to facilitate autocrine and/or paracrine regulation of OXT neurons towards physiologic demands, such as lactation (Moos and Richard, 1989; Neumann et al., 1994) and child birth (Neumann et al., 1996). To exert neuronal effects, locally released OXT binds to local OXTRs, which are expressed within or are juxtaposed to the target region, for example, on synapses, as well as on axons and glial processes (Mitre et al., 2016). Alternatively, OXT could putatively diffuse over longer distances to bind to adjacent OXTRs (Landgraf and Neumann, 2004; Ludwig and Leng, 2006; Mitre et al., 2016). Given that OXT exerts its multiple behavioral effects through its action on several regions of the forebrain and mesolimbic brain, the question of whether other extrahypothalamic projections of OXT neurons may also have a role garners significant importance.

It is also becoming increasingly clear that both anterior and posterior pituitary hormones, traditionally thought of as regulators of single physiological processes, affect multiple bodily systems, either directly or via actions on brain receptors (Zaidi et al., 2018; Abe et al., 2003). Nontraditional actions of OXT include its ability to affect the skeleton, wherein it stimulates bone formation by osteoblasts and modulates the function of bone-resorbing osteoclasts (Sun et al., 2019).

Despite a *corpus* of evidence for the expression of OXT and OXTRs in various brain regions, and their function in regulating central and peripheral actions, such as social behavior and satiety (Sun et al., 2019; Bale et al., 2001), there remains the need for a detailed, sex-specific mapping of the anatomical geography of the OXT and OXTR systems in the brain. Here, we use RNAscope—a cutting-edge technology that detects single RNA transcripts—to create a comprehensive sex-specific atlas of the OXT and OXTR in the mouse brain. We believe that this compendium of OXT and its receptor in concrete brain sites should provide a resource for investigators to study both peripheral and central effects of interrogating OXTRs site—specifically in health and disease. Our identification of brain nuclei with the highest OXT and OXTR transcript density will thus deepen our future understanding of the functional engagement of the central OXT-containing neuronal nodes within a large-scale functional network.

## Results

Mapping autoradiographic studies suggest that the distribution of OXTRs in the brain varies greatly among different rodent species (Dubois-Dauphin et al., 1992; Elands et al., 1988; Insel et al., 1997; Tribollet et al., 1992). Besides mapping the full anatomical distribution of *Oxt* and *Oxtr* by RNAscope, the present study also assessed sex differences in *Oxt* and *Oxtr* distribution. Allowing the detection of single transcripts, RNAscope uses ~20 pairs of transcript-specific double *Z*-probes to hybridize 10-µm-thick whole-brain sections. Preamplifiers first hybridize to the ~28-bp binding site formed by each double *Z*-probe; amplifiers then bind to the multiple binding sites on each preamplifier; and finally, labeled probes containing a fluorescent molecule bind to multiple sites of each amplifier.

RNAscope data were quantified on sections from coded three female and three male mice. Each section was viewed and analyzed using CaseViewer 2.4 (3DHISTECH, Budapest, Hungary) and QuPath v.0.2.3 (University of Edinburgh, UK). The *Atlas for the Mouse Brain in Stereotaxic Coordinates* (Paxinos and Franklin, 2007) was used to identify every nucleus, sub-nucleus, or region, which was followed by manual counting of *Oxt* and *Oxtr* transcripts by two independent observers (VR and AG) in every tenth section using a tag feature. Receptor density was calculated by dividing the transcript number by the area (µm^2^, ImageJ) in every nucleus, sub-nucleus, or region. Photomicrographs were prepared using Photoshop CS5 (Adobe Systems) only to adjust brightness, contrast, and sharpness, and to remove artifacts (i.e., obscuring bubbles).

**Figure 1.**
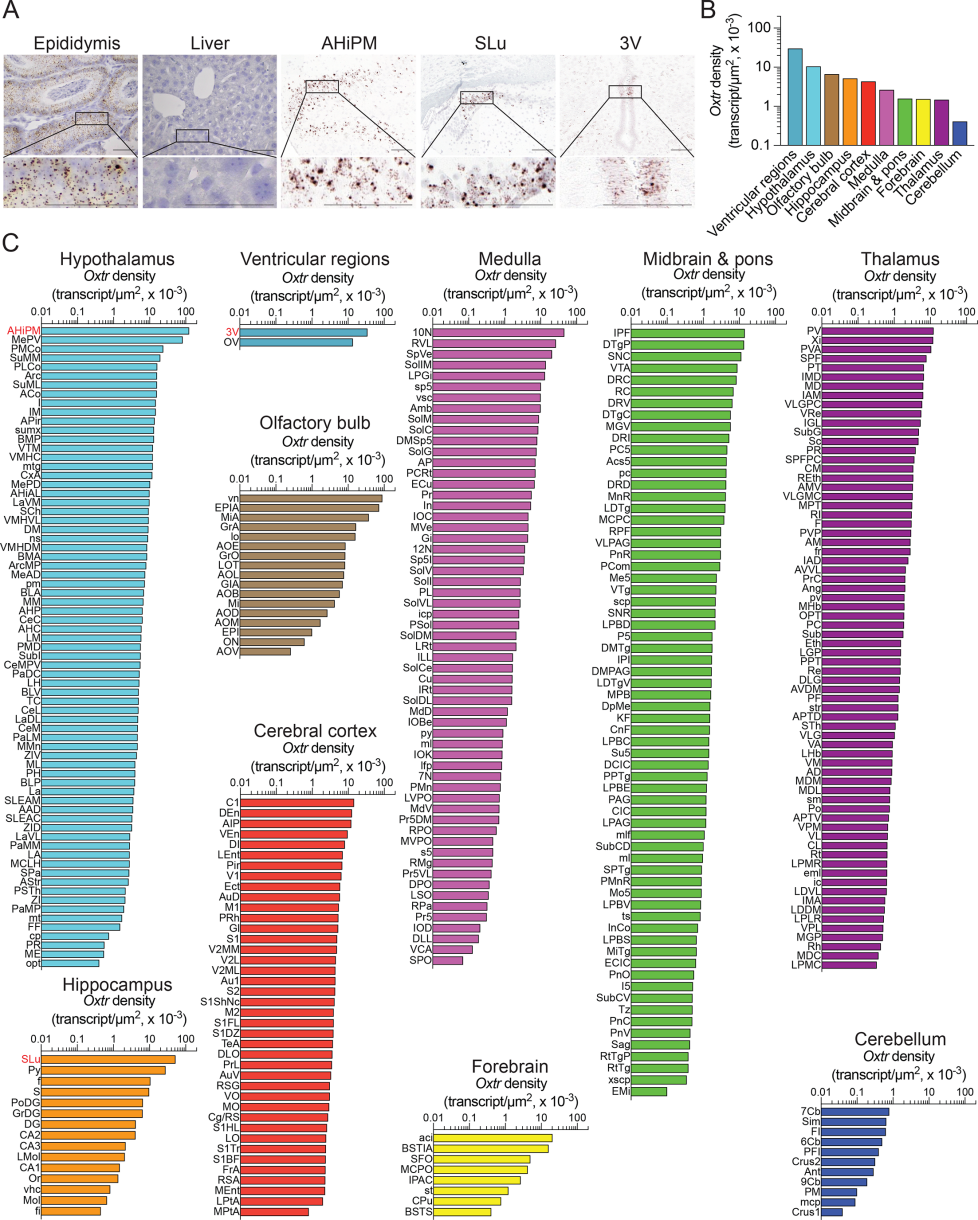
*Oxtr* expression in the male brain. (**A**) RNAscope revealed *Oxtr*-positive transcripts in the epididymis, but not in the liver (positive and negative controls, respectively). Also shown are representative micrographs of the posteromedial part of the amygdalohippocampal area (AHiPM) of the hypothalamus, pyramidal cell layer (Py) of the hippocampus, and the third ventricle (3V) of the ventricular regions. Scale bar: 50 µm. (**B**) *Oxtr* transcript density in the brain regions detected by RNAscope. (**C**) *Oxtr* transcript density in nuclei, sub-nuclei, and regions of the ventricular system, hypothalamus, olfactory bulb, hippocampus, cerebral cortex, medulla, midbrain and pons, forebrain, thalamus, and cerebellum. Figure 1—source data 1.*Oxtr* densities in brain nuclei, subnuclei and regions.

In males, we report the expression of the *Oxtr* in 359 mouse brain nuclei, sub-nuclei, and regions. Probe specificity was established by a positive signal in the epididymis with an absent signal in the liver (negative control) (Figure 1A). Notably, *Oxtr* transcripts were detected bilaterally, with no apparent ipsilateral domination. Transcript density was highest in ventricular regions, followed, in descending order, by the hypothalamus, olfactory bulb, hippocampus, cerebral cortex, medulla, midbrain and pons, forebrain, thalamus, and cerebellum (Figure 1B). Using the RNAscope dataset, we further calculated *Oxtr* density in various brain nuclei, sub-nuclei, and regions. High *Oxtr* transcript densities and counts, respectively, were also noted in several nuclei, sub-nuclei, and regions as follows (Figure 1C): ventricular regions—ependyma of the OV and 3V; hypothalamus—AHiPM for both; olfactory bulb—vn and GrO; hippocampus—Py for both; cerebral cortex—Cl and Pir; medulla—10N and Sp5I; midbrain and pons—IPF and DpMe; forebrain—aci and CPu; thalamus—PV and PVA and cerebellum—Sim for both (see Appendix 1 for nomenclature and Figure 1—figure supplement 1 for transcript count and representative photomicrographs).

**Figure 1—figure supplement 1.**
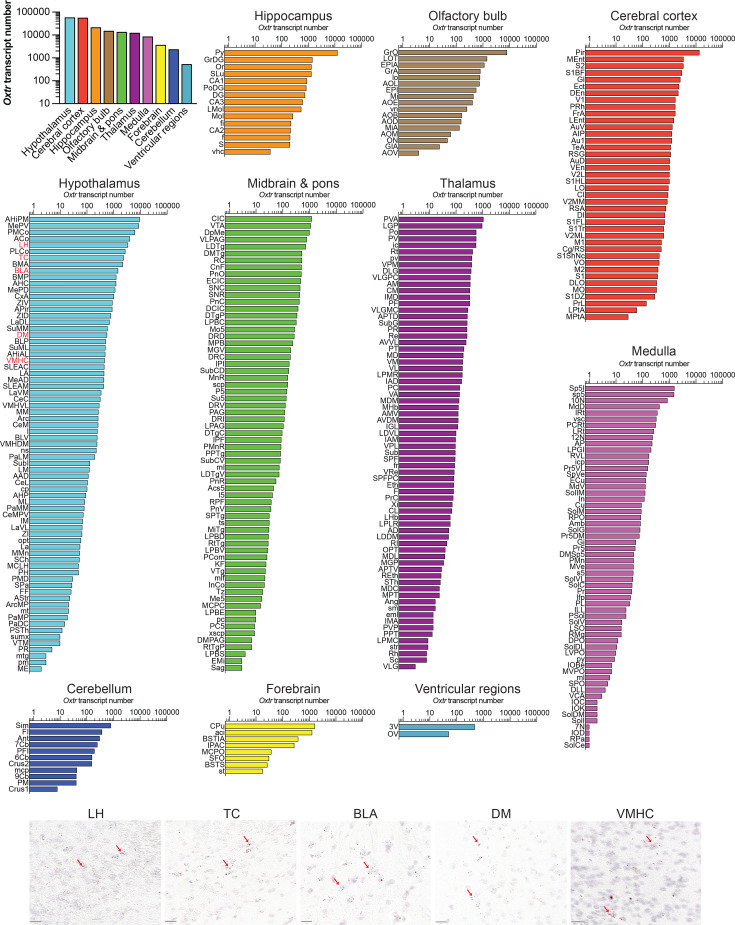
*Oxtr* expression in the male brain. *Oxtr* transcript number in brain regions and in nuclei, sub-nuclei, and regions of the hypothalamus, cerebral cortex, hippocampus, olfactory bulb, midbrain and pons, thalamus, medulla, forebrain, cerebellum, and ventricular system detected by RNAscope. Also shown are representative RNAscope images of several hypothalamic regions, including lateral hypothalamus (LH), tuber cinereum (TC), anterior part of the basolateral amygdaloid nucleus (BLA), dorsomedial hypothalamus (DM), and central part of ventromedial hypothalamic nucleus (VMHC). Note that LH and DM send SNS outflow to both bone and fat tissue. Scale bar: 20 µm. Figure 1—figure supplement 1—source data 1.*Oxtr* transcript numbers in brain nuclei, subnuclei and regions.

**Figure 2.**
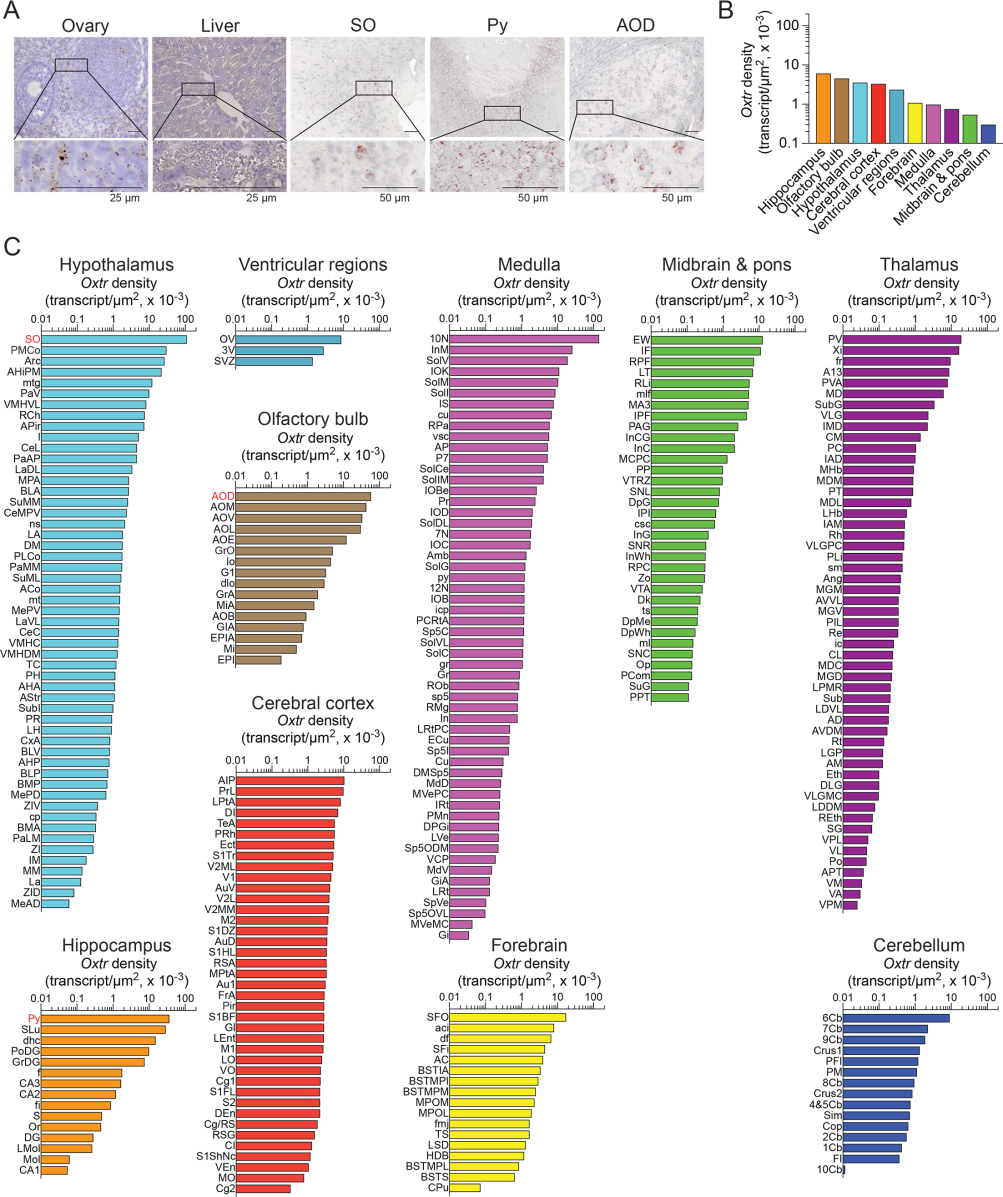
*Oxtr* expression in the female brain. (**A**) Representative micrographs of the supraoptic nucleus (SO) of the hypothalamus, the pyramidal cell layer (Py) of the hippocampus, and anterior olfactory nucleus, dorsal part (AOD) of the olfactory bulb are shown. Ovary and liver served as positive and negative controls, respectively. Scale bar: 50 µm. (**B**) *Oxtr* transcript density in the brain regions detected by RNAscope. (**C**) *Oxtr* transcript density in nuclei, sub-nuclei, and regions of the hippocampus, olfactory bulb, hypothalamus, cerebral cortex, ventricular system, forebrain, medulla, thalamus, midbrain and pons, and cerebellum. Figure 2—source data 1.*Oxtr* densities in brain nuclei, subnuclei and regions.

In females, we report the expression of the *Oxtr* in 301 mouse brain nuclei, sub-nuclei, and regions. Probe specificity was again established by a positive signal in the ovary with no signal in the liver (Figure 2A). Transcript density was highest in the hippocampus, followed, in descending order, by the olfactory bulb, hypothalamus, cerebral cortex, ventricular regions, forebrain, medulla, thalamus, midbrain and pons, and cerebellum (Figure 2B). High *Oxtr* transcript densities and counts, respectively, were also noted in several nuclei, sub-nuclei, and regions as follows (Figure 2B): hippocampus—Py for both; olfactory bulb—AOD and GrO; hypothalamus—SO and PMCo; cerebral cortex—AIP and Pir; ventricular regions—ependyma of the OV and SVZ; forebrain—SFO and aci; medulla—10N for both; thalamus—PV for both; midbrain and pons—EW and PAG and cerebellum—6Cb for both (see Appendix 1 for nomenclature and Figure 2—figure supplement 1 for transcript count and representative photomicrographs).

**Figure 2—figure supplement 1.**
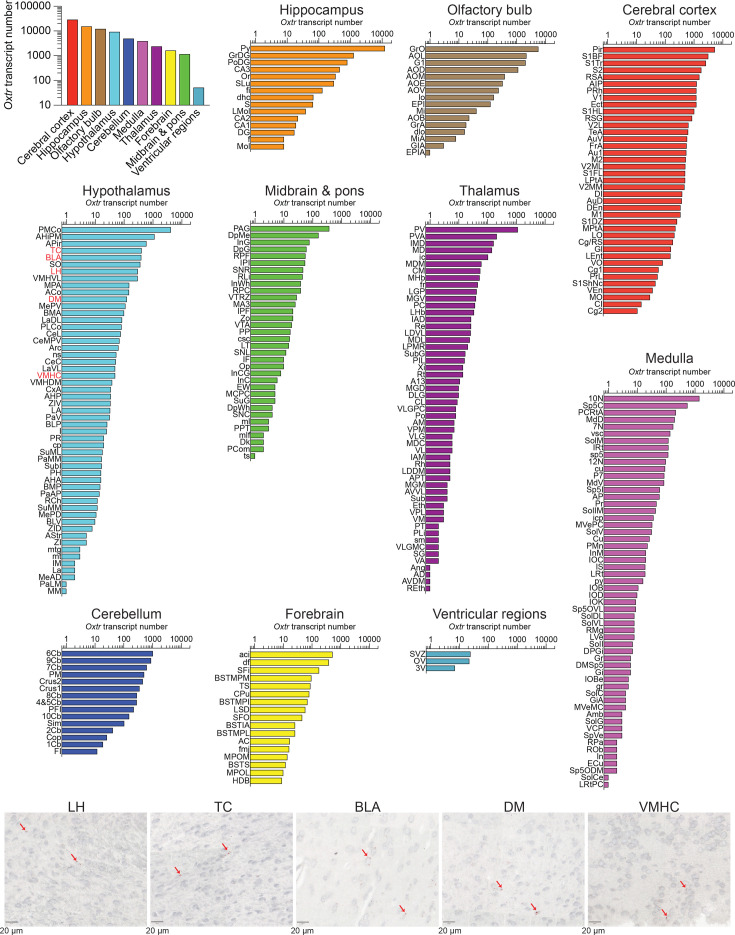
*Oxtr* expression in the female brain. *Oxtr* transcript number in brain regions and in nuclei, sub-nuclei, and regions of the cerebral cortex, hippocampus, olfactory bulb, hypothalamus, cerebellum, medulla, thalamus, forebrain, midbrain, and pons, and ventricular system detected by RNAscope. Representative RNAscope images of the lateral hypothalamus (LH), tuber cinereum (TC), anterior part of the basolateral amygdaloid nucleus (BLA), dorsomedial hypothalamus (DM), and central part of ventromedial hypothalamic nucleus (VMHC) are shown. Scale bar: 20 µm. Figure 2—figure supplement 1—source data 1.*Oxtr* trancript numbers in brain nuclei, subnuclei and regions.

**Figure 3.**
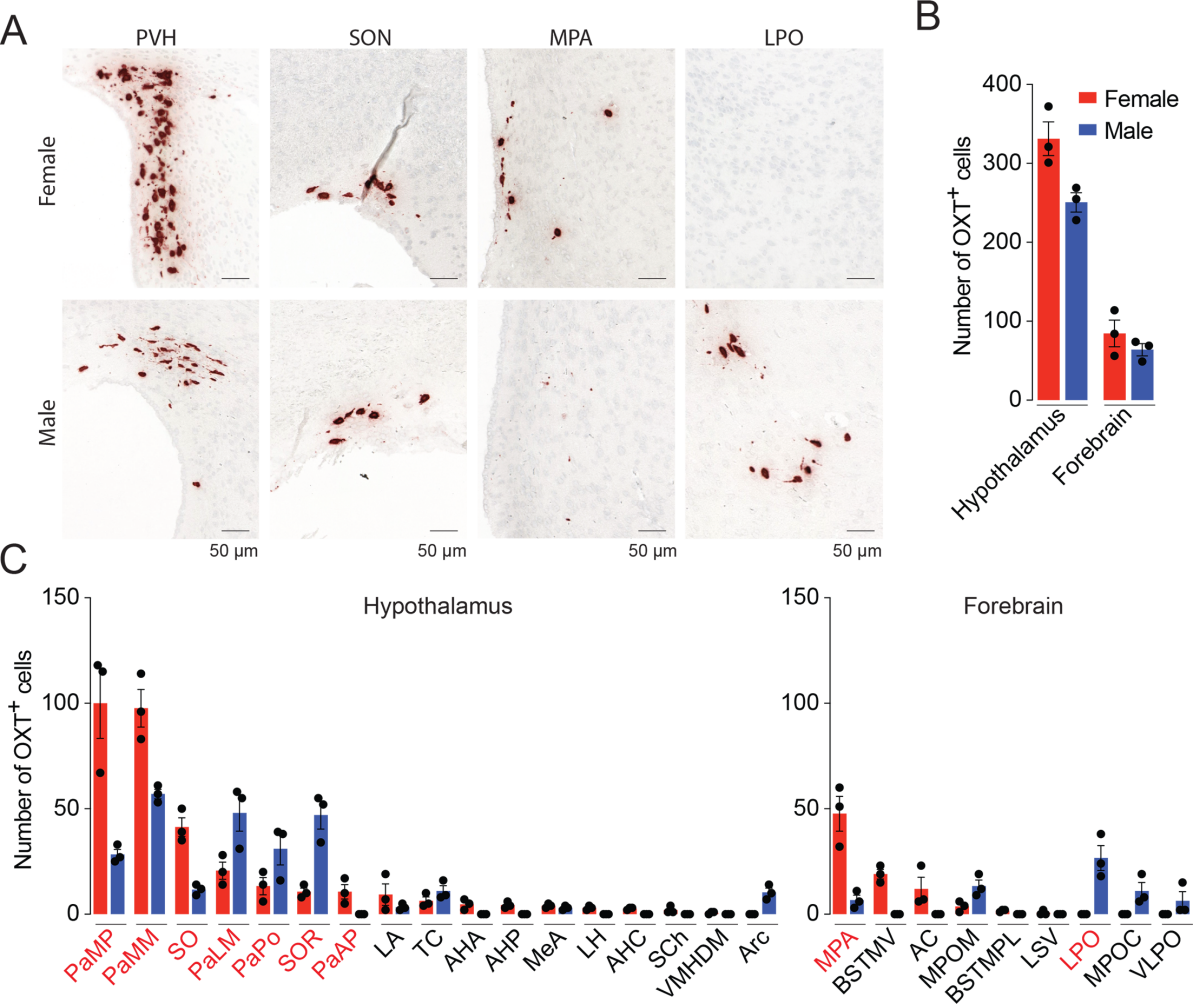
Sex differences in *Oxt* expression in the mouse brain. (**A**) The neurohypophysial hormone OXT is synthesized by magnocellular neurons primarily located in the PVH and SON hypothalamic nuclei. The magnocellular neurons send extended axonal projections into the neurohypophysis where OXT is released into the circulation in response to physiological demands. Therefore, PVH and SON served as positive controls for OXT expression in the brain. MPA: medial preoptic area; LPO: lateral preoptic area of the forebrain. Scale bar: 50 µm. (**B**) Sex differences in the numbers of *Oxt*-expressing neurons in nuclei, sub-nuclei, and regions of the hypothalamus and forebrain detected by RNAscope. (**C**) Total numbers of *Oxt*-expressing neurons in nuclei, sub-nuclei, and regions of the hypothalamus and forebrain of male and female mice. (**D**) *Oxt* transcript density in nuclei, sub-nuclei, and regions of the hypothalamus and forebrain of male and female mice. N = 3, values are shown as means ± SE. Student’s *t*-test. Figure 3—source data 1.Numbers of OXT-positive cells in the hypothalamus and forebrain.

RNAscope also revealed *Oxt* expression in the hypothalamus and forebrain of both male and female mice (Figure 3A and B). High *Oxt* counts were detected in several nuclei, sub-nuclei, and regions of females and males, respectively, as follows (Figure 3C): hypothalamus—PaMP and PaMM and forebrain—MPA and LPO. Overall, the numbers of *Oxt*-expressing cells were markedly greater in female compared to the male mice. That is, we found 22 hypothalamic and forebrain regions with 486 *Oxt*-positive cells in the female mouse. In contrast, there were 15 hypothalamic and forebrain regions containing 308 *Oxt*-positive cells in the male brain. Breaking this down, in the hypothalamus, the number of *Oxt*-positive cells was 372 in the female compared with 228 *Oxt*-positive cells in the male. The number of *Oxt*-positive cells was 114 in the female forebrain compared with 80 *Oxt*-positive cells in the male forebrain.

**Figure 4.**
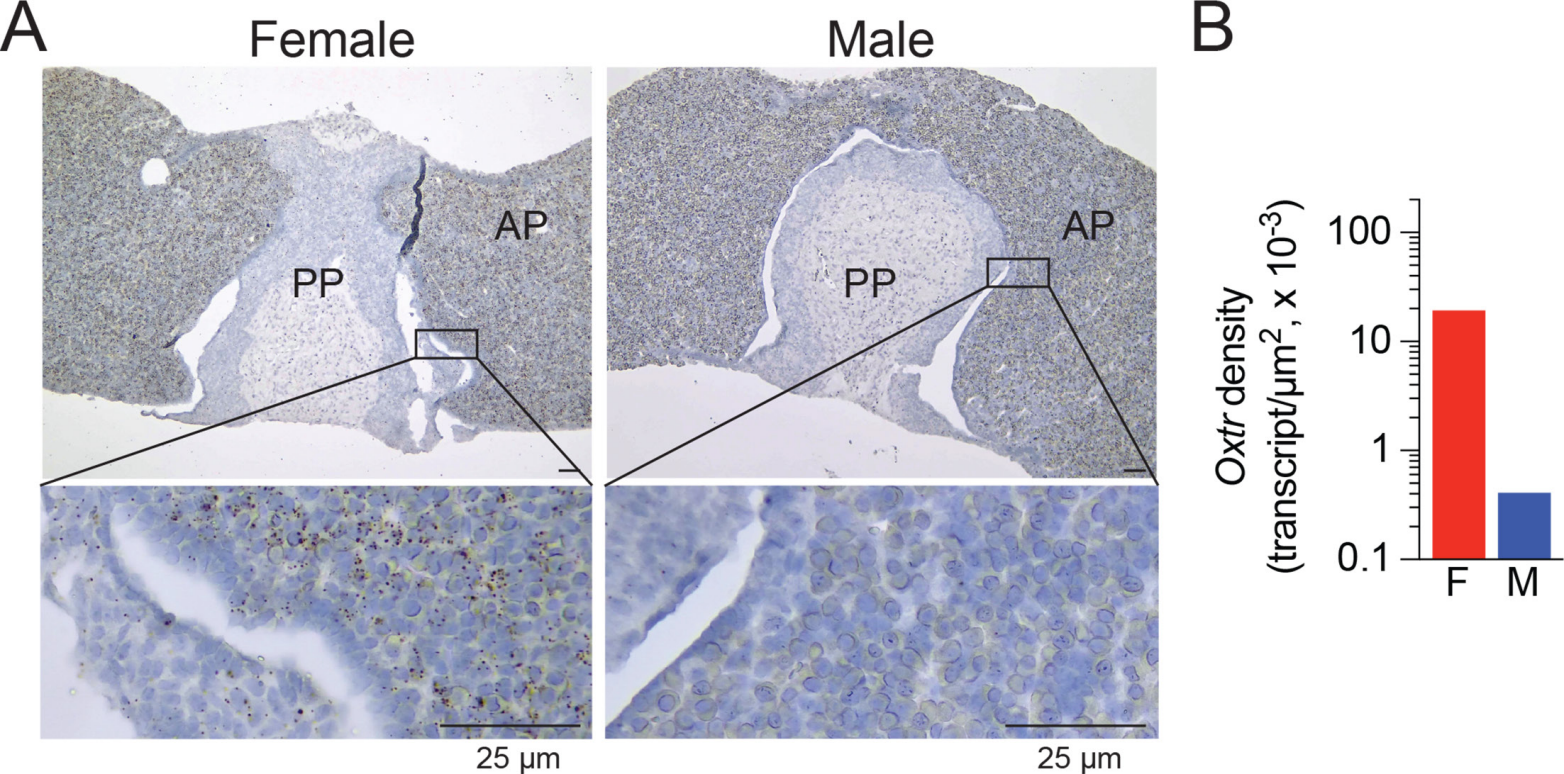
Sex differences in *Oxtr* expression in the pituitary gland. (**A**) Representative photomicrographs showing sex differences in *Oxtr* expression in anterior (AP) and posterior (PP) lobes of the pituitary gland detected by RNAscope. Scale bar: 25 µm. (**B**) Quantification of *Oxtr* transcript density in the pituitary gland of female and male mice (n=3). Figure 4—source data 1.Sex-specific *Oxtr* densities in the pituitary gland.

*Oxtr* expression was also mapped in regions and sub-regions within the hypothalamus (Figure 1 and Figure 1—figure supplement 1). Certain of these hypothalamic sub-regions, such as the lateral hypothalamus (LH) and dorsomedial hypothalamus (DM), send sympathetic nervous system (SNS) outflow to both bone and fat tissue (Ryu et al., 2015; Ryu et al., 2017). Additionally, RNAscope also showed *Oxtr* expression in both anterior and posterior pituitary lobes (Figure 4A), with *Oxtr* transcript density that was markedly higher in the female compared with male mice (Figure 4B).

We also found that six hypothalamic nuclei, sub-nuclei, and regions in male mice displayed overlapping *Oxt* and *Oxtr* transcripts. *Oxtr*/*Oxt* ratios within the same brain site were as follows: 0.70 in the medial parvicellular part of the paraventricular hypothalamic nucleus (PaMP); 1.51 in the medial magnocellular part of the paraventricular hypothalamic nucleus (PaMM); 6.39 in the lateral magnocellular part of the paraventricular hypothalamic nucleus (PaLM); 26.8 in the arcuate nucleus (Arc); 109.50 in the medial amygdaloid nucleus (MeA); 151.13 in the tuber cinereum area (TC), and 222.00 in the lateroanterior hypothalamic nucleus (LA). In contrast, we found three forebrain nuclei, sub-nuclei, and regions in female mice with overlapping *Oxt* and *Oxtr* transcripts. *Oxtr*/*Oxt* ratios within the same brain site were as follows: 1.35 in the anterior commissural nucleus (AC); 3.50 in the medial preoptic nucleus, medial part (MPOM), and 13.0 in the bed nucleus of the stria terminalis, medial division, and posterolateral part (BSTMPL). As with the hypothalamus, *Oxtr*/*Oxt* ratios in ten nuclei, sub-nuclei, and regions were 0.05 in the PaLM; 0.18 in the PaMM; 0.82 in the paraventricular hypothalamic nucleus, anterior parvicellular part (PaAP); 1.74 in the LA; 5.83 in the anterior hypothalamic area, posterior part (AHP); 8.00 in the anterior hypothalamic area, anterior part (AHA); 9.33 in the supraoptic nucleus (SO); 39.0 in the ventromedial hypothalamic nucleus, dorsomedial part (VMHDM); 40.7 in the TC and 75.3 in the lateral hypothalamic area (LH).

## Discussion

Here, we supplement and integrate previous information on OXT and OXTR expression in the murine brain and report, for the first time, abundant OXTR expression in 301 and 359 brain nuclei, sub-nuclei, and regions in females and males, respectively, as well as, importantly, sex-specific *Oxt* and *Oxtr* expression. This report is thus the most comprehensive atlas of brain *Oxt* and *Oxtr* expression at the single transcript level. Expression of both *Oxt* and *Oxtr*, particularly in overlapping hypothalamic sub-nuclei, nuclei, and regions, points to functionally active neuronal nodes within a large-scale OXT-OXTR network in the brain.

It has been reported that cell bodies and dendrites of OXT-producing neurons within the PVH and SON release OXT and AVP within the magnocellular nuclei, where we find the highest *Oxtr/Oxt* colocalization—this suggests an additional, possibly paracrine action of OXT (Ludwig and Leng, 2006; Neumann et al., 1993; Neumann, 2007; Pow and Morris, 1989). Indeed, locally released OXT is involved in pre- and post-synaptic modulation of the electrical activity (Bourque et al., 1993; Shibuya et al., 2000; Kombian et al., 1997). Similar to magnocellular neurons of the PVH, we find that several *Oxt*-producing neuronal populations also overlap with *Oxtr* expression in other hypothalamic sites—hereby termed ‘*Oxtr*/*Oxt* nodes’—these include Arc, MeA, TC, and LA.

It is now well known that central OXT decreases ingestive behavior while OXTR antagonism has the opposing effect in rodents (Liu et al., 2021; Arletti et al., 1990; Blevins et al., 2016; Klockars et al., 2018; Liu et al., 2020; Noble et al., 2014; Ong et al., 2015). Of note is that, in addition to the nucleus of the solitary tract (NTS) (Ong et al., 2015), the *Oxtr*/*Oxt* node in the Arc (and, possibly, Arc-bordering LA) rapidly induces satiety (Fenselau et al., 2017) and suppresses excessive food intake to control ingestive behavior (Inada et al., 2022). In terms of sex-specific ability of OXT to inhibit ingestive behavior, it has been reported that the capacity of OXT to decrease food intake is attenuated in females compared with males, whereas lower OXT doses are effective at reducing food intake in males, and doses that are effective in both sexes reduce consumption for a longer duration in males (Liu et al., 2020). The *Oxtr*/*Oxt* node in the MeA likely explains the paracrine regulation by OXT of male preference for females and their scents (Yao et al., 2017). Thus, the ablation of *Oxtr* in aromatase-expressing neurons of the MeA fully recapitulates the elimination of female preference in males, suggesting that this node is both necessary and sufficient for social recognition (Ferguson et al., 2001). Lastly, the TC is a sheet of gray matter that forms a median eminence (ME) around the base of the pituitary stalk or infundibulum; therefore, the *Oxtr*/*Oxt* node in the TC (and tanycyte) could be important for mediating bidirectional brain–periphery crosstalk by modulating the blood–hypothalamus brain barrier.

Although we detected clear sex differences and similarities in *Oxtr* transcript expression in multiple brain areas, here we will focus on those associated with stress, energy homeostasis, emotional, and affective behaviors. Surprisingly, the highest *Oxtr* transcript density was noted in the ependymal layers of the OV and 3V in both sexes with greater expression density in males and, not surprisingly, in the hypothalamus (Sofroniew, 1983; Swanson and Sawchenko, 1983; Landgraf and Neumann, 2004). In the hypothalamus, the highest density was found in the posteriomedial part of the amygdalohippocampal area (AHiPM) of males compared to that in females. It has been reported that in male mice, ~40% of *Oxtr*-positive neurons of the amygdalohippocampal area (AHi) project to the medial preoptic area (MPOA) (Sato et al., 2020). Activation of these neurons, comprising excitatory projections to the MPOA, enhances exclusively an aggressive, but not parental behavior, towards pups (Sato et al., 2020). Of interest, females display the highest *Oxtr* density in the arcuate nucleus (Arc) compared to that of males. Arc^Vglut2^ neurons have been reported to express the gene encoding *Oxtr* (Fenselau et al., 2017). Given that intra-Arc OXT acutely suppresses food intake and OXT exerts a direct stimulatory effect on Arc-OXTR neurons, it is plausible that the Arc-OXTR-satiety circuit, at least, responding to diet-induced hyperphagia (Maric et al., 2022), is pronounced in female rather than male mice. Indeed, it was demonstrated that female rats and mice display a lower than male level of diet-induced overeating (Maric et al., 2022).

The male olfactory bulb and hippocampus also displayed abundant *Oxtr* transcripts, with the highest density in the vomeronasal nerve (vn) and the pyramidal cell layer of the hippocampus (Py), respectively, in comparison to the female anterior olfactory nucleus, dorsal part (AOD) as well as the same (Py) hippocampal subregion. It has been reported that *Oxtrs* are expressed in the vomeronasal organ, an olfactory sensory structure involved in the detection of non-volatile chemosignals. OXT injection in mice has been shown to reduce pup-induced vomeronasal activity and aggressive behavior (Nakahara et al., 2020). Given vomeronasal activity declines as males grow up from a pup-aggressive state to a non-aggressive parental state, high *Oxtr* expression in the vn might indicate a functional switch from pup-aggressive behavior towards strengthening social and sexual behaviors during adolescence and adulthood. The highest *Oxtr* transcript densities in the AOD, AOM, and AOV of females are consistent with OXT function in the anterior olfactory region, particularly in relation to social cue processing and social recognition (Oettl et al., 2016). As with the hippocampal Py, *Oxtrs* are found in both excitatory and inhibitory pyramidal neurons within the CA2 and CA3 subregions of the hippocampus, suggesting that OXTRs in the Py may have a role in local circuits relating to stress, emotional, and affective behaviors.

In the cortex, we found that agranular insular cortex, posterior part (AIP) of females and claustrum (Cl) of males displayed the highest *Oxtr* transcript density. PVH- and SON-OXT neurons project to a wide range of cortical and limbic structures including AIP, Cl, hippocampus, medial amygdala, and the lateral septum, all of which comprise the social recognition memory circuit (Mitre et al., 2016; Ferguson et al., 2001; Son et al., 2022; Tanimizu et al., 2017; Wang and Zhan, 2022). SON neurons, upon activation by the OXTR, release OXT—a putative paracrine loop. Moreover, OXTRs mediate cardiac sympathetic stimulation through direct PVH projections to the intermediolateral column of the spinal cord (Japundžić-Žigon, 2013). Such reciprocal communications are supported by the studies inferring that affiliative social interactions increase OXT activity, which is followed by an anti-stress response, thus promoting bonding, relaxation, and growth, while reducing cardiovascular and neuroendocrine stress (Grippo et al., 2009; Krause et al., 2011; Lee et al., 2005; Windle et al., 1997; Wsol et al., 2008).

Both males and females had the highest *Oxtr* transcript density in the medullary dorsal motor nucleus of vagus (10N), as has been shown previously in the rat (Raggenbass et al., 1988; Dreifuss et al., 1988; Raggenbass et al., 1987). The OXT-sensitive vagal neurons are mostly preganglionic motor neurons, projecting to the cervical, thoracic, and abdominal visceral areas (Raggenbass et al., 1987). It has also been shown that the microinjection of an OXT antagonist into the 10N blocks the increase in gastric acid secretion and bradycardia induced by electrical stimulation of the PVH—this suggests a role for central OXT in autonomic efferent activity (Rogers and Hermann, 1986).

Finally, we have recently published an atlas of pituitary glycoprotein hormone receptors, namely *Tshr*, *Fshr,* and *Lhcgr*, in more than 400 brain sites (Ryu et al., 2022). Surprisingly, we find a striking overlap in receptor distribution among the four receptors, including the *Oxtr*—with highest transcript levels in the ependymal layer of the third ventricle and olfactory bulb. While the role of olfactory OXTRs in social recognition is well established (Oettl et al., 2016; Sun et al., 2021; Oettl and Kelsch, 2018), the functional significance of OXTRs in the ependymal layer is yet unknown. However, in light of ubiquitous and newly emerging OXTR expression in the brain and peripheral organs, ependymal OXTRs seem to have an important role in gating the bidirectional brain–periphery crosstalk.

Despite higher plasma OXT levels in women than in men (Marazziti et al., 2019), prior, largely immunohistochemistry-based studies failed to identify a sex difference in *Oxt* expression in the brain. Similar numbers of OXT-positive immunoreactive (-IR) neurons were found in the PVH, SON, MPOA, and bed nucleus of stria terminalis (BNST) of prairie, pine, meadow, and montane voles (Wang et al., 1996), PVH and SON of naked mole rats (Rosen et al., 2008), and PVH, MPOA, LH, and anterior hypothalamus (AH) of long-tailed hamsters (Xu et al., 2010). Furthermore, no sex differences were detected in OXT-IR neurons in the PVH, SON, BNST, MeA in several species of non-human primates (Caffé et al., 1989; Wang et al., 1997a; Wang et al., 1997b). There were also no sex differences in *Oxt* mRNA expression in the PVH and SON of the rat (for review, see Dumais and Veenema, 2016). Lastly, there were no sex differences in the number or size of OXT neurons in the PVH and SON in humans (Wierda et al., 1991; Fliers et al., 1985; Ishunina and Swaab, 1999). By contrast, here we establish sex differences in *Oxt* expression in the mouse brain. Both the hypothalamus and forebrain of the females contained visibly more *Oxt*-positive cells compared with males. Whereas as expected, hypothalamic PVH of both sexes had high *Oxt* expression, the medial preoptic area (MPA) of the female forebrain and the lateral preoptic area (LPO) of the male forebrain contained the highest number of *Oxt*-expressing cells.

The neuroanatomical reality of the brain–bone–fat SNS feedback loops suggests coordinated and/or multiple redundant control of bone and fat remodeling (Ryu et al., 2024). We have noted that regions, such as the LH, DM, tuber cenereum area (TC), basolateral amygdaloid nucleus, and others, known to send SNS outflow to both bone and adipose tissues, express the *Oxtr* (Ryu et al., 2015; Ryu et al., 2017). Surprisingly, female mice had visibly fewer *Oxtr* counts in aforementioned sites compared to males, perhaps due to the organizational and activational effects of sex hormones (Kammel and Correa, 2020). This raises the possibility that certain actions of OXT on peripheral tissues, such as on body composition and bone, may also be mediated centrally. Indeed, non-classical actions of OXT include its ability to affect bone remodeling, wherein it stimulates bone formation by osteoblasts and modulates the function of bone-resorbing osteoclasts (Sun et al., 2019). We have also shown that OXT and vasopressin have opposing skeletal actions—effects that may relate to the pathogenesis of bone loss in pregnancy and lactation, and in chronic hyponatremia, respectively (Sun et al., 2019; Sun et al., 2016; Tamma et al., 2009; Tamma et al., 2013). As with fat remodeling, it has been demonstrated that mice deficient in either OXT or OXTRs develop late-onset obesity despite normal ingestive behavior (Takayanagi et al., 2008). Moreover, the increased body weight in OXT knockout mice is accompanied by a 40% increase in abdominal adiposity (Camerino, 2009).

In all, studies on central OXT signaling and its control of reproductive, metabolic, and ingestive functions, and social behaviors occupy the vast majority of the literature. It is our hope that this comprehensive compendium of sex-specific *Oxt* and *Oxtr* expression in the brain will stimulate further investigations by others. In more general terms, the direct mapping of receptor expression in the brain and periphery provides the framework for determining new functions of ancient pituitary hormones and helps refocus at least some in the field towards paradigm-shifting discoveries of non-traditional, multifaceted roles of OXT.

## Methods

### Mice

Adult C57BL/6J mice (~3–4-month-old) were housed in a 12 h:12 h light:dark cycle at 22 ± 2°C with ad libitum access to water and regular chow. All procedures were approved by the Mount Sinai Institutional Animal Care and Use Committee and are in accordance with Public Health Service and United States Department of Agriculture guidelines. Ethical approval for all experimental procedures wasobtained from the appropriate Institutional Review Board under protocol number PROTO202100038.

### RNAscope

Brains and pituitary glands were collected from male and female mice (n=3) for RNAscope. Briefly, mice were anesthetized with isoflurane (2–3% in oxygen; Baxter Healthcare, Deerfield, IL) and transcardiacally perfused with 0.9% heparinized saline followed by 10% neutral buffered formalin (NBF). Brains were extracted, sectioned into 0.5 cm (whole pituitary and adrenal glands) thick slices, and post-fixed in 10% NBF for 24 h, dehydrated, and embedded into paraffin. Coronal sections were cut at 5 μm with every tenth section mounted onto ~60 slides with three sections on each slide. This method allowed us to cover the entire brain and eliminate the likelihood of counting the same transcript twice. Sections were air-dried overnight at room temperature and stored at 4°C until required.

Detection of mouse *Oxt* and *Oxtr* was performed separately on paraffin sections using Advanced Cell Diagnostics (ACD) RNAscope 2.5 LS Multiplex Reagent Kit (#322100) and two RNAscope 2.5 LS probes, namely Mm-OXT (#493178) and Mm-OXTR (#412178). Epididymis/ovary and liver served as positive and negative controls for *Oxtr*, respectively. As with OXT, magnocellular cells of the PVH and SON served as positive controls while the brain from *Oxt* knockout mouse served as a negative control.

Slides were baked at 60°C for 1 h, deparaffinized, incubated with hydrogen peroxide for 10 min at room temperature, pretreated with Target Retrieval Reagent (#322001) for 20 min at 100°C and with Protease III for 30 min at 40°C. Probe hybridization and signal amplification were performed as per the manufacturer’s instructions for chromogenic assays.

Following the RNAscope assay, the slides were scanned at ×20 magnification, and the digital image analysis was successfully validated using the CaseViewer 2.4 (3DHISTECH, Budapest, Hungary) software. The same software was employed to capture and prepare images for the figures in the article. Images of control tissues were taken using a Leica DM 1000 microscope. Detection of *Oxtr*- and *Oxt*-positive cells was also performed using the QuPath-0.2.3 (University of Edinburgh, UK) software based on receptor intensity thresholds, size, and shape.

### Statistical analysis

Data were analyzed by two-tailed Student’s *t*-test and one-way repeated measures analysis of variance followed by Holm–Sidak’s or Bonferroni’s least significant difference post hoc tests using GraphPad Prism 10 (Boston, MA). Significance was set at p<0.05. For simplicity and clarity, exact test results and exact p values are not presented.

## Data Availability

All data generated or analyzed during this study are included in the manuscript and supporting files; source data files have been provided for Figures 1—4, Figure 1—figure supplement 1 and Figure 2—figure supplement 1.

## Additional files

MDAR checklist

## Appendix 1

**Appendix 1—table 1.** Glossary of the brain nuclei, sub-nuclei, and regions.

Olfactory bulb	
AOB	accessory olfactory bulb
AOD	anterior olfactory nucleus, dorsal part
AOE	anterior olfactory nucleus, external part
AOL	anterior olfactory nucleus, lateral part
AOM	anterior olfactory nucleus, medial part
AOV	anterior olfactory nucleus, ventral part
EPI	external plexiform layer of the olfactory bulb
EPIA	external plexiform layer of the accessory olfactory bulb
GIA	glomerular layer of the accessory olfactory bulb
GrA	granule cell layer of the accessory olfactory bulb
GrO	granular cell layer of the olfactory bulb
lo	lateral olfactory tract
LOT	nucleus of the lateral olfactory tract
Mi	mitral cell layer of the olfactory bulb
MiA	mitral cell layer of the accessory olfactory bulb
ON	olfactory nerve layer
vn	vomeronasal nerve
Cerebral cortex	
AIP	agranular insular cortex, posterior part
Au1	primary auditory cortex
AuD	secondary auditory cortex, dorsal area
AuV	secondary auditory cortex, ventral area
Cl	claustrum
Cg/RS	cingular/retrosplenial cortex
DEn	dorsal endopiriform nucleus
DI	dysgranular insular cortex
DLO	dorsolateral orbital cortex
Ect	ectorhinal cortex
FrA	frontal association cortex
GI	granular insular cortex
LEnt	lateral entorhinal cortex
LO	lateral orbital cortex
LPtA	lateral parietal association cortex
M1	primary motor cortex
M2	secondary motor cortex
MEnt	medial entorhinal cortex
MO	medial orbital cortex
MPtA	medial parietal association cortex
Pir	piriform cortex
PRh	perirhinal cortex
PrL	prelimbic cortex
RSA	retrosplenial agranular cortex
RSG	retrosplenial granular cortex
S1	primary somatosensory cortex
S1BF	primary somatosensory cortex, barrel field
S1DZ	primary somatosensory cortex, dysgranular region
S1FL	primary somatosensory cortex, forelimb region
S1HL	primary somatosensory cortex, hindlimb region
S1ShNc	primary somatosensory cortex, shoulder/neck region
S1Tr	primary somatosensory cortex, trunk region
S2	secondary somatosensory cortex
TeA	temporal association cortex
V1	primary visual cortex
V2L	secondary visual cortex, lateral area
V2ML	secondary visual cortex, mediolateral area
V2MM	secondary visual cortex, mediomedial area
VEn	ventral endopiriform nucleus
VO	ventral orbital cortex
Forebrain	
AC	anterior commissural nucleus
aci	anterior commissure, intrabulbar part
BSTIA	bed nucleus of the stria terminalis, intraamygdaloid division
BSTMPL	bed nucleus of the stria terminalis, medial division, posterolateral part
BSTMV	bed nucleus of the stria terminalis, medial division, ventral part
BSTS	bed nucleus of stria terminalis, supracapsular part
IPAC	interstitial nucleus of the posterior limb of the anterior commissure
LPO	lateral preoptic area
LSV	lateral septal nucleus, ventral part
MCPO	magnocellular preoptic nucleus
MPA	medial preoptic area
MPOC	medial preoptic nucleus, central part
MPOM	medial preoptic nucleus, medial part
SFO	subfornical organ
st	stria terminalis
VLPO	ventrolateral preoptic nucleus
Hippocampus	
CA1	field ca1 of hippocampus
CA2	field ca2 of hippocampus
CA3	field CA3 of hippocampus
DG	dentate gyrus
f	fornix
fi	fimbria of the hippocampus
GrDG	granular layer of the dentate gyrus
LMol	lacunosum moleculare layer of the hippocampus
Mol	molecular layer of the dentate gyrus
Or	oriens layer of the hippocampus
PoDG	polymorph layer of the dentate gyrus
Py	pyramidal tract
S	subiculum
SLu	stratum lucidum, hippocampus
vhc	ventral hippocampal commissure
Thalamus	
AD	anterodorsal thalamic nucleus
AM	anteromedial thalamic nucleus
AMV	anteromedial thalamic nucleus, ventral part
Ang	angular thalamic nucleus
APTD	anterior pretectal nucleus, dorsal part
APTV	anterior pretectal nucleus, ventral part
AVDM	anteroventral thalamic nucleus, dorsomedial part
AVVL	anteroventral thalamic nucleus, ventrolateral part
CL	centrolateral thalamic nucleus
CM	central medial thalamic nucleus
DLG	dorsal lateral geniculate nucleus
eml	external medullary lamina
Eth	ethmoid thalamic nucleus
F	nucleus of the fields of Forel
fr	fasciculus retroflexus
IAD	interanterodorsal thalamic nucleus
IAM	interanteromedial thalamic nucleus
ic	internal capsule
IGL	intergeniculate leaf
IMA	intramedullary thalamic area
IMD	intermediodorsal thalamic nucleus
LDDM	laterodorsal thalamic nucleus, dorsomedial part
LDVL	laterodorsal thalamic nucleus, ventrolateral part
LGP	lateral globus pallidus
LHb	lateral habenular nucleus
LPLR	lateral posterior thalamic nucleus, laterorostral part
LPMC	lateral posterior thalamic nucleus, mediocaudal part
LPMR	lateral posterior thalamic nucleus, mediorostral part
MD	mediodorsal thalamic nucleus
MDC	mediodorsal thalamic nucleus, central part
MDL	mediodorsal thalamic nucleus, lateral part
MDM	mediodorsal thalamic nucleus, medial part
MGP	medial globus pallidus (entopeduncular nucleus)
MHb	medial habenular nucleus
MPT	medial pretectal nucleus
OPT	olivary pretectal nucleus
PC	paracentral thalamic nucleus
PF	parafascicular thalamic nucleus
Po	posterior thalamic nuclear group
PPT	posterior pretectal nucleus
PR	prerubral field
PrC	precommissural nucleus
PT	paratenial thalamic nucleus
pv	periventricular fiber system
PV	paraventricular thalamic nucleus
PVA	paraventricular thalamic nucleus, anterior part
PVP	paraventricular thalamic nucleus, posterior part
Re	reuniens thalamic nucleus
REth	retroethmoid nucleus
Rh	rhomboid thalamic nucleus
RI	rostral interstitial nucleus of medial longitudinal fasciculus
Rt	reticular thalamic nucleus
Sc	scaphoid thalamic nucleus
sm	stria medullaris of the thalamus
SPF	subparafascicular thalamic nucleus
SPFPC	subparafascicular thalamic nucleus, parvicellular part
STh	subthalamic nucleus
str	superior thalamic radiation
Sub	submedius thalamic nucleus
SubG	subgeniculate nucleus
VA	ventral anterior thalamic nucleus
VL	ventrolateral thalamic nucleus
VLG	ventral lateral geniculate nucleus
VLGMC	ventral lateral geniculate nucleus, magnocellular part
VLGPC	ventral lateral geniculate nucleus, parvicellular part
VM	ventromedial thalamic nucleus
VPL	ventral posterolateral thalamic nucleus
VPM	ventral posteromedial thalamic nucleus
VRe	ventral reuniens thalamic nucleus
Xi	xiphoid thalamic nucleus
Hypothalamus	
AAD	anterior amygdaloid area, dorsal part
ACo	anterior cortical amygdaloid nucleus
AHA	anterior hypothalamic area, anterior part
AHC	anterior hypothalamic area, central part
AHiAL	amygdalohippocampal area, anterolateral part
AHiPM	amygdalohippocampal area, posteromedial part
AHP	anterior hypothalamic area, posterior part
APir	amygdalopiriform transition area
Arc	arcuate hypothalamic nucleus
ArcMP	arcuate hypothalamic nucleus, medial posterior part
AStr	amygdalostriatal transition area
BLA	basolateral amygdaloid nucleus, anterior part
BLP	basolateral amygdaloid nucleus, posterior part
BLV	basolateral amygdaloid nucleus, ventral part
BMA	basolateral amygdaloid nucleus, anterior part
BMP	basomedial amygdaloid nucleus, posterior part
CeC	central amygdaloid nucleus, capsular part
CeL	central amygdaloid nucleus, lateral division
CeM	central amygdaloid nucleus, medial division
CeMPV	central amygdaloid nucleus, medial posteroventral part
cp	cerebral peduncle, basal part
CxA	cortex-amygdala transition zone
DM	dorsomedial hypothalamic nucleus
FF	fields of Forel
I	intercalated nuclei of the amygdala
IM	intercalated amygdaloid nucleus, main part
LA	lateroanterior hypothalamic nucleus
La	lateral amygdaloid nucleus
LaDL	lateral amygdaloid nucleus, dorsolateral part
LaVL	lateral amygdaloid nucleus, ventrolateral part
LaVM	lateral amygdaloid nucleus, ventromedial part
LH	lateral hypothalamic area
LM	lateral mammillary nucleus
MCLH	magnocellular nucleus of the lateral hypothalamus
ME	median eminence
MeA	medial amygdaloid nucleus, anterior part
MeAD	medial amygdaloid nucleus, anteriodorsal part
MePD	medial amygdaloid nucleus, posterodorsal part
MePV	medial amygdaloid nucleus, posteroventral part
ML	medial mammillary nucleus, lateral part
MM	medial mammillary nucleus, medial part
MMn	medial mammillary nucleus, median part
mt	mammillothalamic tract
mtg	mammillotegmental tract
ns	nigrostriatal bundle
opt	optic tract
PaAP	paraventricular hypothalamic nucleus, anterior parvicellular part
PaDC	paraventricular hypothalamic nucleus, dorsal cap
PaLM	paraventricular hypothalamic nucleus, lateral magnocellular part
PaMM	paraventricular hypothalamic nucleus, medial magnocellular part
PaMP	paraventricular hypothalamic nucleus, medial parvicellular part
PaPo	paraventricular hypothalamic nucleus, posterior part
PH	posterior hypothalamic area
PLCo	posterolateral cortical amygdaloid nucleus
pm	principal mammillary tract
PMCo	posteromedial cortical amygdaloid nucleus (C3)
PMD	premammillary nucleus, dorsal part
PR	prerubral field
PSTh	parasubthalamic nucleus
SCh	suprachiasmatic nucleus
SLEAC	sublenticular extended amygdala, central part
SLEAM	sublenticular extended amygdala, medial part
SO	supraoptic nucleus
SOR	supraoptic nucleus, retrochiasmatic part
SPa	subparaventricular zone of the hypothalamus
Subl	subincertal nucleus
SuML	supramammillary nucleus, lateral part
SuMM	supramammillary nucleus, medial part
sumx	supramammillary decussation
TC	tuber cinereum area
VMHC	ventromedial hypothalamic nucleus, central part
VMHDM	ventromedial hypothalamic nucleus, dorsomedial part
VMHVL	ventromedial hypothalamic nucleus, ventrolateral part
VTM	ventral tuberomammillary nucleus
ZI	zona incerta
ZID	zona incerta, dorsal part
ZIV	zona incerta, ventral part
Midbrain and pons	
Acs5	accessory trigeminal nucleus
CIC	central nucleus of the inferior colliculus
CnF	cuneiform nucleus
DCIC	dorsal cortex of the inferior colliculus
DMPAG	dorsomedial periaqueductal gray
DMTg	dorsomedial tegmental area
DpMe	deep mesencephalic nucleus
DRC	dorsal raphe nucleus, caudal part
DRD	dorsal raphe nucleus, dorsal part
DRI	dorsal raphe nucleus, interfascicular part
DRV	dorsal raphe nucleus, ventral part
DTgC	dorsal tegmental nucleus, central part
DTgP	dorsal tegmental nucleus, pericentral part
ECIC	external cortex of the inferior colliculus
EMi	epimicrocellular nucleus
I5	intertrigeminal nucleus
InCo	intercollicular nucleus
IPF	interpeduncular fossa
IPI	interpeduncular nucleus, intermediate subnucleus
KF	Ko¨lliker-Fuse nucleus
LDTg	laterodorsal tegmental nucleus
LDTgV	laterodorsal tegmental nucleus, ventral part
LPAG	lateral periaqueductal gray
LPBC	lateral parabrachial nucleus, central part
LPBD	lateral parabrachial nucleus, dorsal part
LPBE	lateral parabrachial nucleus, external part
LPBS	lateral parabrachial nucleus, superior part
LPBV	lateral parabrachial nucleus, ventral part
MCPC	magnocellular nucleus of the posterior commissure
Me5	mesencephalic trigeminal nucleus
MGV	medial geniculate nucleus, ventral part
MiTg	microcellular tegmental nucleus
ml	medial lemniscus
mlf	medial longitudinal fasciculus
MnR	median raphe nucleus
Mo5	motor trigeminal nucleus
MPB	medial parabrachial nucleus
P5	peritrigeminal zone
PAG	periaqueductal gray
PC5	parvicellular motor trigeminal nucleus
pc	posterior commissure
PCom	nucleus of the posterior commissure
PMnR	paramedian raphe nucleus
PnC	pontine reticular nucleus, caudal part
PnO	pontine reticular nucleus, oral part
PnR	pontine raphe nucleus
PnV	pontine reticular nucleus, ventral part
PPTg	pedunculopontine tegmental nucleus
RC	raphe cap
RPF	retroparafascicular nucleus
RtTg	reticulotegmental nucleus of the pons
RtTgP	reticulotegmental nucleus of the pons, pericentral part
Sag	sagulum nucleus
scp	superior cerebellar peduncle (brachium conjunctivum)
SNC	substantia nigra, compact part
SNR	substantia nigra, reticular part
SPTg	subpedencular tegmental nucleus
Su5	supratrigeminal nucleus
SubCD	subcoeruleus nucleus, dorsal part
SubCV	subcoeruleus nucleus, ventral part
ts	tectospinal tract
Tz	nucleus of the trapezoid body
VLPAG	ventrolateral periaqueductal gray
VTA	ventral tegmental area
VTg	ventral tegmental nucleus
xscp	decussation of the superior cerebellar peduncle
Medulla	
7N	facial nucleus
10N	dorsal motor nucleus of vagus
12N	hypoglossal nucleus
Amb	ambiguus nucleus
AP	area postrema
Cu	cuneate nucleus
DLL	dorsal nucleus of the lateral lemniscus
DMSp5	dorsomedial spinal trigeminal nucleus
DPO	dorsal periolivary region
ECu	external cuneate nucleus
Gi	gigantocellular reticular nucleus
icp	inferior cerebellar peduncle (restiform body)
ILL	intermediate nucleus of the lateral lemniscus
In	intercalated nucleus of the medulla
IOBe	inferior olive, subnucleus B of medial nucleus
IOC	inferior olive, subnucleus C of medial nucleus
IOD	inferior olive, dorsal nucleus
IOK	inferior olive, cap of Kooy of the medial nucleus
IRt	intermediate reticular nucleus
lfp	longitudinal fasciculus of the pons
LPGi	lateral paragigantocellular nucleus
LRt	lateral reticular nucleus
LSO	lateral superior olive
LVPO	lateroventral periolivary nucleus
MdD	medullary reticular nucleus, dorsal part
MdV	medullary reticular nucleus, ventral part
ml	medial lemniscus
MVe	medial vestibular nucleus
MVPO	medioventral periolivary nucleus
PCRt	parvicellular reticular nucleus
PL	paralemniscal nucleus
PMn	paramedian reticular nucleus
Pr	prepositus nucleus
Pr5	principal sensory trigeminal nucleus
Pr5DM	principal sensory trigeminal nucleus, dorsomedial part
Pr5VL	principal sensory trigeminal nucleus, ventrolateral part
PSol	parasolitary nucleus
py	pyramidal tract
RMg	raphe magnus nucleus
RPa	raphe pallidus nucleus
RPO	rostral periolivary region
RVL	rostroventrolateral reticular nucleus
s5	sensory root of the trigeminal nerve
SolC	nucleus of the solitary tract, commissural part
SolCe	nucleus of the solitary tract, central part
SolDL	solitary nucleus, dorsolateral part
SolDM	nucleus of the solitary tract, dorsomedial part
SolG	nucleus of the solitary tract, gelatinous part
SolI	nucleus of the solitary tract, interstitial part
SolIM	nucleus of the solitary tract, intermediate part
SolM	nucleus of the solitary tract, medial part
SolV	solitary nucleus, ventral part
SolVL	nucleus of the solitary tract, ventrolateral part
sp5	spinal trigeminal tract
Sp5I	spinal trigeminal nucleus, interpolar part
SPO	superior paraolivary nucleus
SpVe	spinal vestibular nucleus
VCA	ventral cochlear nucleus, anterior part
vsc	ventral spinocerebellar tract
Cerebellum	
6Cb	6th Cerebellar lobule
7Cb	7th Cerebellar lobule
9Cb	9th Cerebellar lobule
Ant	anterior lobe cerebellum
Crus1	crus 1 of the ansiform lobule
Crus2	crus 2 of the ansiform lobule
FI	flocculus
mcp	middle cerebellar peduncle
PFI	paraflocculus
PM	paramedian lobule
Sim	simple lobule
Ventricular zones	
3V	3rd ventricle
OV	olfactory ventricle (olfactory part of lateral ventricle)
